# CIDF-WSN: A Collaborative Interest and Data Forwarding Strategy for Named Data Wireless Sensor Networks

**DOI:** 10.3390/s21155174

**Published:** 2021-07-30

**Authors:** Muhammad Salah ud din, Muhammad Atif Ur Rehman, Byung-Seo Kim

**Affiliations:** 1 Department of Electronics & Computer Engineering, Hongik University, Sejong City 30016, Korea; salah_udin@mail.hongik.ac.kr (M.S.u.d.); atif_r@mail.hongik.ac.kr (M.A.U.R.); 2 Department of Software and Communications Engineering, Hongik University, Sejong City 30016, Korea

**Keywords:** named data networking, wireless sensor networks, internet of things, information-centric networks, single radio WSNs, layered based WSNs, environmental monitoring, agriculture monitoring, crops monitoring

## Abstract

Recent years have witnessed the huge popularity of Information-Centric Networking (ICN) and its realization as Named Data Networking (NDN) in the context of wireless sensor networks (WSNs). The participating nodes in WSNs are usually equipped with a single radio interface. The existing solutions lack in providing the efficient next forwarder selection in NDN-based single radio WSNs. In this work, we propose a collaborative Interest and Data Forwarding (CIDF-WSN) Strategy for Named Data Wireless Sensor Networks. CIDF-WSN develop a Neighbor Information Base (NFIB) which enables the node to select the optimal next-hop relay in Interest packet forwarding. An efficient Interest packet processing mechanism assisted by the Interest Cache Table (ICT) is provided to avoid Interest packets loss and frequent re-transmissions. In addition, CIDF-WSN also provides a robust Data packet transfer mechanism accompanied by the Temp Cache Table (TCT) to avoid Data packet losses and to ensure well-timed content delivery. Simulation results reveal that CIDF-WSN outperforms the recently published works in terms of Interest satisfaction rate, total energy consumption, Data retrieval delays, and communication overhead.

## 1. Introduction

The rapid proliferation of the Internet of Things (IoT) has gained significant attention in recent times as it transforms the modern world into a vast interconnected network of things (also referred to as objects). These objects have been equipped with the capability to generate data, exchange information, and interact with the environment. Wireless sensor networks (WSNs) are deemed as a promising component of IoTs as their environmental monitoring capability accompanied by the data collection, storage, and processing capacity make them fully compliant with the expectations of IoT. These networks have been utilized in a variety of applications such as smart grids [1], smart agriculture monitoring [2], smart health [3], smart home [4], environmental monitoring [5], battlefield surveillance [6], and smart building monitoring [7]. WSNs cannot operate in isolation and have to be connected to the internet to enable the monitoring and controlling authorities to approach the underlying sensor nodes for data acquisition [8].

Communication between the nodes in conventional WSNs is carried out through an address-based communication model [9]. However, this architecture poses various challenges (discussed in Section 2).

To address the intrinsic restraints of address-based architecture while taking into account the requirements of WSNs, a potential flavor of Information-centric networking architecture referred to as Named Data Networking (NDN) has garnered significant attention specifically in terms of IoT. Unlike the traditional address-based networks, NDN considers the content as a first-class citizen of the network [10]. The consumers only specify what they want rather than the exact location where they expect the query to be entertained [11]. Each data unit is self-identifying and self-authenticating and may acquire by the authorized consumer by providing a persistent, distinctive, and location-independent name. Moreover, NDN architecture provides native support to in-network caching, mobility, and multicasting [9].

NDN was originally developed for the communication between the devices at an internet scale where the devices are usually equipped with multiple radio interfaces. In contrast, the WSNs are usually supplied with a single radio transceiver and all the transmissions and receptions are carried out via that interface. Several efforts have been devoted to the literature to investigate how to effectively utilize the NDN in WSNs [12] and how NDN naturally fits the WSNs compared with the conventional address-based model. Most of the schemes advocate the use of NDN for WSNs in terms of routing and forwarding [13], naming [14], Data caching [15], and security [16] among others. However, these schemes lack in providing a mechanism for potential forwarder selection for efficient content delivery in single radio WSNs.

In this paper, we exploit the single radio WSN nodes in layered-based WSNs and propose CIDF-WSN, an efficient collaborative Interest and Data packet forwarding mechanism. CIDF-WSN develops a neighbor information base (NFIB) table which adapts a list of potential virtual faces–next-hop node Ids, based on their associated cost and enable the consumer to select the optimal next-hop forwarder node in its Interest packet forwarding phase. In order to update the cost associated with each virtual face entry of NFIB, we propose robust downstream Interest processing and upstream Interest processing mechanisms. In downstream interest processing, the downstream node in the lower layer overhears the transmission of the upper layer forwarder and update its associated virtual face cost in the NFIB table. The updated NFIB allows the node to designate the optimal node in future Interest forwarding. In addition to NFIB cost maintenance, the upstream Interest processing procedure allows the immediate neighbors of the nominated node to take part in communication and play a pivotal role in minimizing the redundant broadcasts when the designated node does not have information about the potential next hop or fail to further forward the Interest packets. Furthermore, the CIDF-WSN’s unique Data forwarding procedure reduces the incoming Data packet loss due to unpredictable channel variations and upholds the QoS of the network. Finally, the cache tables, such as Temp Cache Table (TCT) and Interest Cache Table (ICT), developed by the CIDF-WSN play an indispensable role and decrease the overall Interest and Data packet losses.

The main contributions of our proposed work are summarized as follows:

1. **Layered Architecture:** CIDF-WSN puts forward a layer-based architecture for Interest and Data packet forwarding for resource-constrained Named Data Internet of Things (IoT).
2. **NFIB for single radio WSN nodes:** We extend the existing NDN architecture and propose a new table named NFIB which enable the node to select the optimal next forwarder in Interest forwarding.
3. **Interest packet processing:** CIDF-WSN develop an efficient Interest processing mechanism to avoid Interest packets loss and to update the cost associated with the NFIB entries.
4. **Data packet recovery:** CIDF-WSN presents a robust Data packet recovery mechanism to avoid Data packet loss when the designated lower layer node in the breadcrumb path fails to forward a packet.
5. **Interest Cache Table (ICT) and Temp Cache Table (TCT):** we devise an Interest Cache Table (ICT) and Temp Cache Table (TCT) to avoid transmission(s) losses.

Through the extensive NDNSim [17] based simulation study, we demonstrated that CIDF-WSN outperforms recently developed schemes for NDN-based WSNs, by reducing the total energy consumption, data retrieval delays, and communication overhead.

The rest of this paper is organized as follows: Section 2 outlines challenges in address-based communication architecture, an overview of NDN and its benefits for WSNs are provided in Section 3. Related work is discussed in Section 4. A detailed description of the CIDF-WSN is provided in Section 5. Section 6 is devoted to a performance evaluation study and finally, Section 7 concludes the paper.

## 2. Challenges in Address-Based Communication Architecture

The conventional host-centric IP-based architecture faces several challenges, especially in the domain of resource-constrained IoTs. Some of the major challenges in address-based architecture are summarized as follows.

1. **Scalability:** The current WSNs deployment utilizes the conventional address-based solution(s) which may induce the efficiency and scalability issues. For instance, extracting the PH value of soil from a single sensor in the agricultural field requires the address and type information of each sensor (i.e., the mapping of address with the content). Therefore, managing the enterprise level deployment of nodes becomes a significantly complex as well as time-consuming operation.
2. **Data Unavailability:** Considering the low battery and low memory constraints of WSN nodes, the Data unavailability issue may arise. To cope with data unavailability problems, an in-network caching-like solution is required. Unfortunately, the conventional IP architecture provides no support for in-network caching [18].
3. **End-to-End connectivity:** In most IoT applications, the consumers are interested in updated Data rather than the location of the producer of that specific Data item [19]. For instance, a user requires the temperature information of certain area (e.g., XYZ) is Interested in the temperature value irrespective of the identity of the temperature-sensing node at that location.
4. **Mobility Support:** There is a lack of native mobility support in IP and to overcome mobility constraint(s), IP utilizes several additional protocols to support the mobility of nodes in dynamic environments.

## 3. NDN Overview and Its Benefits in WSNs

**Figure 1:**
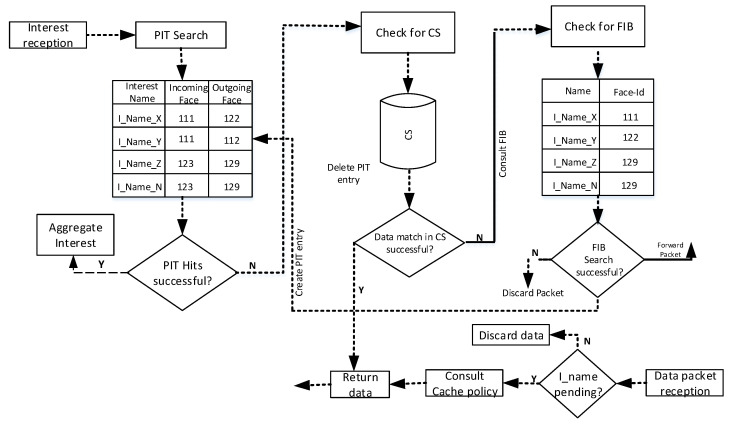
NDN communication architecture.

The idea of NDN was initially proposed by Van Jacobson in [20] and is based on a content-centric networking model. The proposed architecture presents a robust and efficient communication model which is built on Interest and Data packets. The consumer node forwards the Interest packet to retrieve his required content object. On receiving the Interest packet, the corresponding nodes (if they have the requested content in cache) deliver the desired content to the requester by following the chain of PIT entries of the nodes in the reverse path to reach the requester node. The detailed NDN communication process is visualized in Figure 1.

Each NDN node carries the following Data structures: (1) Pending Interest table (2) Content store (CS), and (3) Forwarding Information Base (FIB). The Interest and Data packet forwarding is carried out by consulting the PIT, FIB, and CS. The PIT table records the forwarded Interests, the CS caches the incoming Data packets to fulfill future requests, and the FIB relays the Interest packet(s) towards the potential provider node.

The core features of NDN, such as in-network caching, requests aggregation, and content level security, match with the WSNs applications and efficiently handle the underlying constraints. Some of the benefits that NDN can offer in the domain of WSNs are defined as follows:

- **Ease of application development:** In WSNs the consumer usually demands the Data or content regardless of the location or identifier of the producer node. The consumer application may demand the Data in an information-centric manner irrespective of the physical location of producing entity.
- **Data availability:** The enchanting in-network caching feature of NDN overcomes the problem of information loss due to unavailability of the node which may happen due to several uncertainties, such as battery depletion and sudden hardware or software failure in resource-restrained WSNs.
- **Ease of Data retrieval:** The consumer simply requests the content by issuing an Interest packet that carries the name of the desired content, whereas any receiving node which has already cached the requested content replies with the Data packet regardless of the actual producer of the content. In the network of a large number of wireless sensor nodes, the hierarchical naming provided by the NDN enables easier request/Data aggregation and accelerates the search and information retrieval time. Moreover, the name-based routing mechanism resolves the issue of address space scarcity which may arise in assigning the unique addresses to the huge number of WSN nodes.
- **Scalability:** NDN inherently supports scalability and allows multiple nodes to take part in the Data forwarding process. It greatly improves the quality of communication with an increase in the number of nodes in the given area via intrinsic forwarding procedures.

Information-centric WSNs correspond to the category of WSNs which mainly focuses on information retrieval rather than establishing the point-to-point (P2P) communication setup between the objects. In this regards several efforts have been devoted to literature to provide seamless and efficient information sharing.

## 4. Related Work

Table 1 offers a quick glance at some NDN-based schemes developed for WSNs. The authors of [21] discuss CCN-WSN—a lightweight, flexible content-centric networking protocol for wireless sensor networks. In this work, the authors modified several aspects of CCNx in order to meet the resource-constrained nature of WSNs. The proposed protocol modifies the message format and presents a flexible naming scheme for WSNs. In [22], energy-efficient Interest forwarding in NDN-based wireless sensor networks was proposed. The authors proposed two forwarding modes named flooding mode and directive mode for NDN-enabled WSNs. The proposed work mainly focuses on the energy consumption of WSNs and designs of various energy conservation mechanisms such as flexible mode shift, flooding scope control, broadcast storm avoidance, packet suppression, and energy weight factors to preserve and equalize the energy consumption of nodes.

In [23], BLOOGO—Bloom filter based GOssip algorithm for wireless NDN—was proposed. BLOOGO aims to disseminate messages in the entire network with a miniaturized frequency of transmissions. In BLOOGO, the node only relays the message if its neighborhood is not involved in the sending list. The authors performed the neighbors’ relationship comparison by utilizing the bloom filter. Choi et al. [24] proposed an in-network caching effect on optimal energy consumption in content-centric networking. The authors consider various hardware technologies, content popularity, and the total number of downloads in an hour to compute the minimum energy consumption. The genetic algorithm was proposed to find the efficient cache position. The authors claimed that the energy proportional caching, as well as sufficient storage capacity, is very essential to gain energy efficiency in CCN. In [25], multi-source Data retrieval in IoT via named Data networking was proposed for retrieving the Data from several Data sources in NDN-based IoT.A realistic cascading model for multi-sink WSNs was proposed in [28], which mainly focuses on avoiding the cascading failures via placement of multiple sinks in the network.

Authors in [26] developed a novel wireless recharging scheme for NDN-based hierarchical WSN architecture. In this work, the network is partitioned into several sections with one cluster head amongst each section. An energy Interest packet is forwarded in the network to collect the battery level information of the nodes and each node replies with its battery level upon receipt of the Interest. The received energy information is utilized in the selection of section heads.

In [29], the authors discuss the NDN-based routing and forwarding schemes in wireless ad-hoc networks. Due to severe challenges, such as mobility and limited resource nodes in these networks, reactive flooding is employed to discover the potential providers, instead of proactive routing. In [30], the effects of node mobility on spatial network cascading failures are provided. In [31], a routing protocol inspired by the directed diffusion [32] protocol was proposed. The scheme was coupled with multiple anti-collision timers to fulfill periodical monitoring applications’ requirements. In [33], Information-centric networking in the IoT: experiments with NDN in the wild was proposed. The proposed scheme lacks in consideration of flooding scope control, therefore, the proposed work may underperform in resource-restrained WSNs.

In [34], the authors employed the smart building use case and designed a push-based naming scheme to control the Data packets broadcasts in the network. The scheme is mainly composed of a hierarchical namespace for IoT devices, a hierarchical namespace for the content(s), and to support the emergencies a minor modification to the Data packets is done. The proposed naming scheme lacks in providing the pull-based communication for the IoT. In [35], the authors proposed a pull-based hierarchical naming scheme for IoT-based smart homes. The main components of the proposed scheme involved “/homeID, task class, task type, task sub-type, and location”. This proposed scheme covers action as well as sensing tasks; however, it lacks in providing the security mechanism to the critical subcomponents of the above-mentioned components. In addition, the proposed mechanism may not be efficient for transient IoT contents and may generate redundant packet transmissions in the network.

Sobia et al. [36] proposed a hierarchical and flat-based hybrid naming (HFHN) scheme for the use case of smart buildings. In this work, the Interest packet’s hierarchical components are composed of the domain name, the geographic location, and the task, whereas the hash of the device name is the flat component. To secure the payload in the Data packet, the cryptographic hash was computed. The authors consider the static as well as mobile nodes in the network to evaluate their proposed naming schemes.

## 5. CIDF-WSN

In this section, to elucidate the features of the CIDF-WSN, we first provide the motivation behind our proposed scheme and some network assumptions. Subsequent to that, a detailed explanation of CIDF-WSN is provided.

### 5.1. Motivation

The rapid development of IoT-based technologies has revamped almost every industry including the agriculture sector. The IoT revolution has revamped the conventional agriculture methods with new technologies such as “WSNs” to reduce the environmental impact on crops, attain large production, maintain high quality with minimum labor cost. The WSNs play a backbone role in the agriculture sector, as the sensor nodes sense the environmental conditions such as temperature, humidity, soil moisture level, soil pH, light conditions, and carbon dioxide, etc., and transfer the readings to the monitoring station. These aforementioned readings highly affect the productivity of the crop and require well-timed delivery at the control center to yield better growth. This is because any change in these parameters may directly affect the growth of underlying crop and may require immediate attention (e.g., The 1-degree Celsius increase in the seasonal temperature may lower the overall productivity of rice crop by 9%, while the same increase may lower the productivity of corn crop by 10%) [37]. Therefore, we take into consideration the above-mentioned environmental conditions which have substantial importance in attaining the fine-quality final product.

The conventional WSNs-based agricultural monitoring systems employ the traditional address-based mechanisms which may induce scalability and efficiency issues in the network. For instance, a user requiring the temperature information from a sensor node in the monitoring region requires complete knowledge of all the deployed sensor nodes along with their data type. This procedure makes the overall management of large-scale networks significantly complex and time-consuming. Moreover, in single-channel WSNs, most of the schemes lack in providing efficient next forwarder selection, Interest, and Data Packet recovery mechanisms. Thus, the main motivation of the proposed scheme is to put forward a robust NDN-based IoT-based agricultural monitoring system that eliminates the aforementioned constraints and in the meantime fulfills the fundamental requirements of the agricultural monitoring system in order to yield better growth.

### 5.2. System Model and Network Assumptions

**Figure 2:**
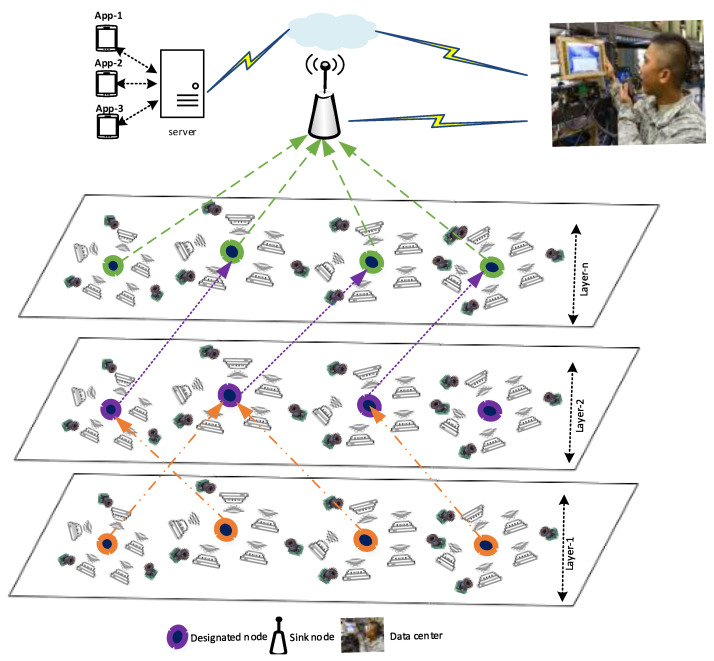
CIDF-WSN system architecture.

The CIDF-WSN system is composed of several heterogeneous sensor nodes that are deployed in the Region of Interest (RoI). The concept of heterogeneity is defined in terms of the nodes’ application type and their available resources, such as storage, battery, and computation resources, among others. The region is further partitioned into several layers and each layer is composed of multiple nodes, as depicted in Figure 2. It is assumed that: (I) each node has the information about the neighboring nodes(s) via Hello packets exchange, as proposed by [27]. (II) The nodes are static, and each node is equipped with a GPS module to acquire its location coordinates. (III) Each node has information about the hosting layer.

### 5.3. CIDF-WSN Operation

Before diving deep into the operational details of the CIDF-WSN, we first shed light on the two main data structures of the NDN forwarding daemon—FIB and PIT—and why they need to be tuned for efficient operation in WSNs. Later, we discuss how the CIDF-WSN ensures effective forwarding in the resource-constrained environment where the nodes are usually equipped with a single radio interface.

The FIB is utilized to forward the Interest packets towards the potential data source that resembles the requested content. As discussed earlier, the FIB table holds the list of outgoing interfaces, and the incoming Interest packets are forwarded towards the potential face by consulting with FIB. The PIT incorporates the information about the Interest that is forwarded towards the upstream (e.g., potential data source) nodes and waiting for the response. Upon receiving an Interest packet, the router first enquires its PIT to ensure whether the same name Interest packet has already been forwarded or not. If the PIT entry already exists, the router adds the face identifier of the incoming Interest packet. Contrarily, the router checks its FIB and selects the face with minimum route cost to forward the Interest packet. Once the forwarding completes, the router inserts the new outgoing face entry in the PIT table (containing the incoming Face address and outgoing Face address of the forwarded Interest packet) representing the forwarded Interest packet. Finally, the Data packets arrive on the reverse path guided by the matching PIT entries.

The aforementioned procedure is utilized for the communication between the devices on the internet where the routers are mostly equipped with an array of interfaces [38]. However, in WSNs the nodes are usually equipped with a single radio interface and the transmissions and receptions of Interest and Data packets are carried out through the same interface. In such networks, the FIB table may contain only a single interface entry, and the selection of optimal interface while having only one candidate interface is meaningless as the nodes have to utilize the same interface to perform Interest and Data transmissions. Therefore, we propose a new neighbor-forwarding information base (NFIB) for single interface equipped WSNs nodes. The NFIB allows the node to select the potential next hop in the presence of a single radio interface. The NFIB maintains a list of virtual faces (potential next-hop node Ids) with their associated cost that assists the node to select the optimal virtual face—representing the next-hop node Id, heading towards the potential Data source. Once the face selection is finalized, the corresponding Interest packet is forwarded to the selected virtual face and the Interest entry is stored in the PIT.

**Figure 3:**
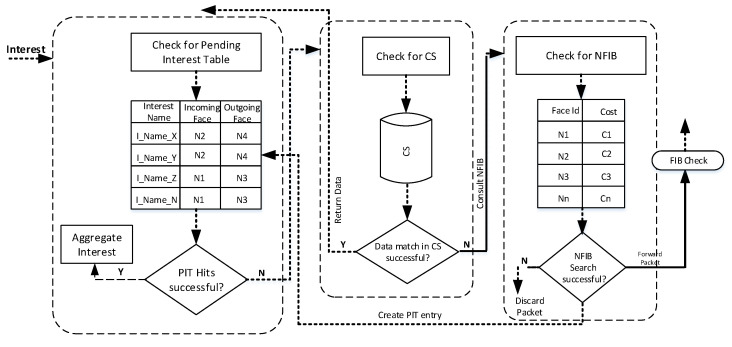
NFIB-based next-hop selection.

It is worth mentioning here that the existing FIB Data structure is used to forward the Interest packet towards the face selected from NFIB on AdHoc Interface. The complete procedure of NFIB-based next-hop selection is depicted in Figure 3, whereas the detailed description of NFIB development is provided in Section 5.3.1.

#### 5.3.1. NFIB Development and Cost Maintenance

The NFIB is comprised of a collection of virtual faces (e.g., potential next-hop node Ids) and their associated cost. An NFIB entry with a certain prefix denotes that given an Interest packet with that prefix, a possible source(s) of requested data can be reached through the face provided by the virtual face Id(s) in the NFIB entry. The associated cost can be computed based on the current conditions of the node, such as workload, number of successful transmissions by the node, available resources, and residual energy reserve, by adopting suitable multi-criteria decision-making (MCDM) procedure [39] and it is out of the scope of the proposed work. All the entries of NFIB are organized in decreasing order of their associated cost and the cost is updated by the lower layer node when the designated upper-layer node further retransmits the Interest. It is to be noted that the virtual face which has a high associated cost will be selected as a potential next-hop relay.

The detailed description of NFIB cost maintenance and the Interest processing mechanism is presented as follows.

#### 5.3.2. Interest Packet Processing

When the interest packet arrives at the designated node, the following Interest packet processing mechanisms, such as Normal Mode Interest Packet processing and recovery mode Interest packet processing, can be executed.

**Figure 4:**
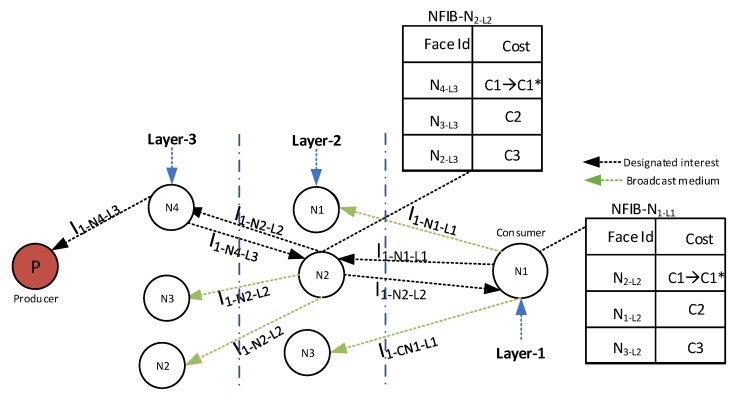
Downstream Interest packet processing.

**Figure 5:**
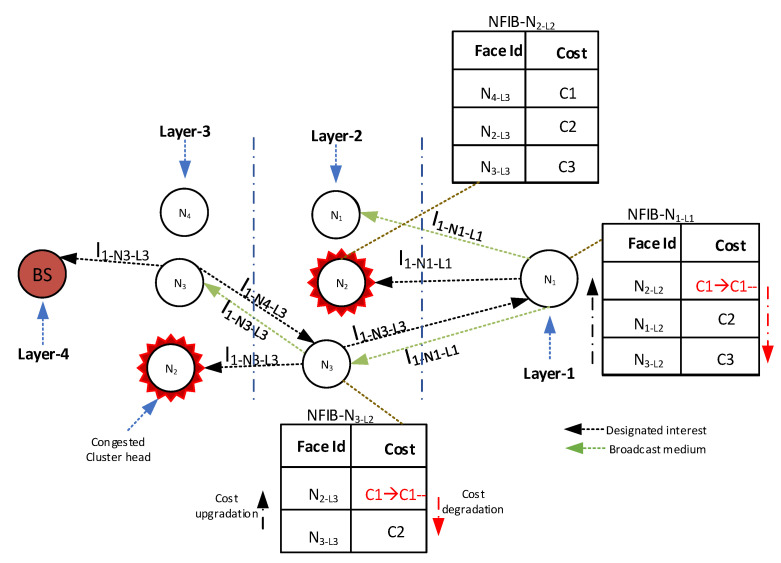
Upstream Interest packet processing.

1. **Normal Mode Interest Packet Processing:** The Normal Mode Interest Packet Processing occurs during stable network conditions. When the lower-layer node forwards the Interest packet to the designated upper-layer node, the packet is also received by the immediate neighbors of the designated node due to the shared medium. The immediate neighboring nodes temporarily store the Interest packet in their ICT (discussed in Section 5.3.4) and associated forwarder timer (the forwarder timer is computed based on the node’s residual energy and distance from the boundary of the layer; the node closer to the boundary of its host layer and high residual energy reservoir has a low forwarder timer value ) with the received packet to avoid the packet losses, in case of designated node failure. Once the Interest reception is finalized, the further Interest processing involves (a) Normal Mode Downstream Interest packet processing and (b) Normal Mode Upstream Interest packet processing.
  (a) **Normal Mode Downstream Interest packet processing:** In the Normal Mode Downstream Interest packet processing, when the upper-layer designated forwarder forwards the Interest packet further, the forwarded Interest packet is also received by the downstream forwarder node (in the lower layer) due to the shared medium. When the downstream node receives the forwarded interest, it increases the number of successful transmission parameters of the upstream forwarder and updates the associated virtual face cost of the upstream forwarder node in the NFIB table by utilizing the cost updating mechanism. The updated NFIB table allows the node to select an upstream relay that has the highest associated cost for the future interest forwarding process.
  (b) **Normal Mode Upstream Interest packet processing:** In the Normal Mode Upstream Interest packet processing, upon the designated node Interest packet transmission, the immediate neighbors of the designated node (in the upper layer) also receive the Interest packet. Upon receipt, these immediate neighbor nodes stop their forwarder timer and purge their ICT to avoid cache pollution and to vacate the ICT to accommodate future Interest requests.
  The complete procedure of Normal Mode Downstream Interest packet processing and Normal Mode Upstream Interest packet processing is shown in Figure 4.
  Consider a scenario where the node N1 of layer 1 ($N1_{L1}$) requests some Data from the producer by forwarding an Interest ($I_{1-N1-L1}$), as shown in Figure 4.
  The $N1_{L1}$ forwards the Interest packet toward the selected virtual face, i.e., N2 of layer 2 ($N2_{L2}$). The neighboring nodes of $N2_{L2}$ ($N1_{L2}$, and $N3_{L2}$) also receive the Interest packet as they fall in the transmission radius of $N1_{L1}$. These neighboring nodes temporarily store the received Interest packet in their ICT and associate the forwarder timer with the Interest packet. The $N2_{L2}$ verifies its node Id against the designated Id presented in the Interest packet and forwards the Interest towards the designated virtual face, i.e., N4 of layer 3 ($N4_{L3}$), by consulting its NFIB. Due to the shared wireless medium, the Interest packet forwarded by the $N2_{L2}$ towards the $N4_{L3}$ is also received by the downstream $N1_{L1}$; therefore, upon receiving the Interest packet, the $N1_{L1}$ (executes the Normal Mode Downstream Interest packet processing) increments the number of successful transmissions of $N2_{L2}$ and updates the associated cost of the face ($N2_{L2}$) in its NFIB (e.g., $C1^{*}$ in the Figure 4 denotes the updated cost of face $N2_{L2}$). Whereas, the neighboring nodes of $N2_{L2}$ (e.g., $N1_{L2}$, and $N3_{L2}$) execute the Normal Mode Upstream Interest packet processing to purge their ICT and stops the forwarder timer.
2. **Recovery Mode Interest packet mechanism:** The wireless channel may never remain consistently reliable and it confronts severe fluctuations due to path losses, capture effect, and fading. As a result, the forwarded Interest may fail to reach the potential provider. The consumer node may resend the Interest packet as it has not received any response against the transmitted Interest. The frequent Interest packet re-transmissions may induce congestion and collisions in the network. To this end, we devise an efficient mechanism referred to as recovery mode. The recovery mode involves two types of processing and is described as follows.
  (a) **Recovery Mode Downstream Interest packet processing:** In Recovery Mode Sownstream Interest packet processing, if the designated node fails to further transmit the Interest packet, the immediate neighbors of the designated node continue the transmissions. As the Interest packet is also received by the immediate neighbors of the forwarder node, these nodes store the Interest packet in ICT and associate the forwarder timer to the received Interest packet. Therefore, on nominated forwarder failure, the immediate neighbor facing the destined provider forwards the Interest packet upon the expiration of the forwarder timer in order to avoid the Interest packet loss. When the neighbor node of the designated node forwards the Interest packet, the downstream forwarder node located in the lower layer overhears the packet, updates the number of successful Interest transmission parameter, and computes the costs of virtual faces in NFIB accordingly.
  (b) **Recovery Mode Upstream Interest packet processing:** As discussed earlier, the medium is shared and the forwarded Interest is also received by other immediate neighbors of the designated node. The nodes (which cache the forwarded Interest packet in the ICT), upon receiving the same forwarded Interest packet, purge their ICT entry and stop their forwarder timer to avoid the ICT redundancy and allocate the space for the future Interest packets.
  The example scenario for both aforementioned mechanisms is depicted in Figure 5 and described as follows.
  As shown in Figure 5, the Interest packet is received successfully at the designated node $N_{2-L2}$ in the upper layer. Due to the shared medium, the Interest packet is also received by the immediate neighboring nodes of the designated node (e.g., $N_{1-L2}$, $N_{3-L2}$). The consumer downstream node ($N_{1-L1}$) overhears the transmission of $N_{2-L2}$ to update the associated cost of $N_{2-L2}$ in the NFIB table. If for certain reasons (e.g., lack of currently available resources, congestion, hardware failure, etc.) the $N_{2-L2}$ fails to forward the request, the forwarder timer of the immediate neighbor expires. To avoid the broadcast storm and ensure the directive forwarding, since the $N_{3-L2}$ is also facing towards the provider, it forwards the Interest packet towards the upstream node, i.e., $N_{2-L3}$. The $N_{1-L1}$ also receives the Interest packet and upon reception, the $N_{1-L1}$ perform the cost computation and update the NFIB table (as shown in the Figure 5) to ensure the improved performance for future transmissions.This process continues until the Interest packet successfully reaches the provider node.

#### 5.3.3. Data Packet Transfer

Once the Interest packet reaches the producer node, the producer node verifies the Interest packet authenticity, assembles the Data packet of the requested content, and forwards it towards the consumer. In the conventional NDN, the Data packet follows the reverse path of PIT entries maintained in the PIT table of downstream nodes. This means that the Data are forwarded back to the consumer node by following the breadcrumb path of the received Interest packet. Following the breadcrumb path, the Data delivery at the consumer end may fail due to poor channel conditions. To cope with these constraints, CIDF-WSN offers a robust Data packet forwarding mechanism to avoid Data packet loss. The Data packet forwarding includes (1) Normal Mode Data Forwarding and (2) Recovery Mode Data Forwarding.

**Figure 6:**
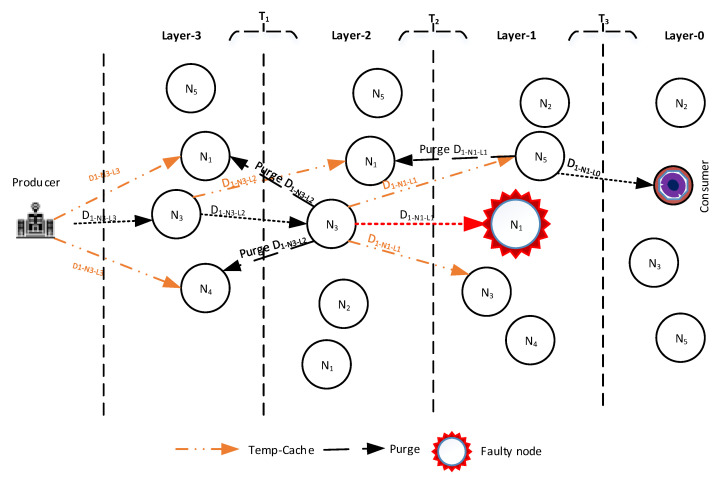
Data packet transfer.

The detailed operational description of the above mentioned Data Forwarding modes assisted by the illustrative example depicted in Figure 6 is as follows.

1. **Normal Mode Data packet forwarding:** The Normal Mode Data packet forwarding refers to the Data packet forwarding towards the lower-layer downstream virtual face (node) from where the Interest request arrives. Upon the arrival of the Data packet, the designated downstream node verifies the received Data packet and forwards it towards its designated lower-layer Forwarder. The immediate neighboring nodes of the designated node in the same layer also store the Data packet in their TCT (discussed in Section 5.3.4) and associate the forwarder timer with the received Data packet to avoid the data packet losses when the designated node fails to forward the Data packet towards the consumer. Upon designated node Data forwarding, the immediate neighboring nodes also receive the Data packet. These immediate neighbors stop their forwarder timer and purge the Data packet from their respective TCT to avoid redundancy. This process continues until the Data packet arrives at the provider node.
2. **Recovery Mode Data forwarding:** Recovery Mode Data forwarding corresponds to the immediate neighbors of the downstream node which may take part in Data forwarding during disastrous conditions. If the designated forwarder does not receive or fails to retransmit the Data packet in the reverse path towards the consumer, the immediate neighboring nodes of the designated node directing towards the consumer act as the backup nodes and forward the Data packet incorporating their TCT. The information of the next forwarder downstream node—in the breadcrumb path—is maintained in the PIT table of the failed designated node while the immediate neighbors of the failed node do not have that entry in their PIT table. In this case, the potential immediate neighboring node upon expiration of forwarder timer utilizes TCT and broadcasts the Data packet towards the lower-layer nodes. Upon receipt of the Data packet, the nodes in the lower layer compare the name of received Data with their outgoing requests record in the PIT. The node with a matching PIT entry with the incoming Data name immediately forwards the Data packet towards its designated downstream layer node. The remaining neighboring nodes which have the same entry of forwarded Data packet in their TCT stop their forwarder timer and purge their cache upon further retransmission by the lower-layer designated node. This process significantly improves the interest satisfaction ratio, circumvents redundant transmissions, and avoids long transmission delays.

The complete Data packet forwarding process is presented in Figure 6. The figure elucidates that once the Interest arrives, the Producer node verifies the Interest, assembles the requested content in the Data packet, and forwards it in the reverse path. In transmission phase $T_{0}$ (as shown in Figure 6), the producer forwards the Data packet towards the $N_{3-L3}$. To avoid the Data packet loss, the neighboring nodes of the $N_{3-L3}$ (i.e., $N_{1-L3}$, $N_{4-L3}$), cache the Data packet in their TCT and associate the forwarder timer with the received Data packet name. The $N_{3-L3}$ retransmit the Data packet towards the lower-layer $N_{3-L2}$ in transmission $T_{1}$. As the network conditions are stable, upon receipt, the $N_{3-L2}$ again retransmit the Data towards the $N_{1-L1}$ in $T_{2}$. During $T_{2}$, the $N_{1-L3}$ and $N_{4-L3}$ stop their respective timers and purge their caches to avoid cache pollution. Whereas, the $N_{1-L2}$ acts as the backup node and caches the incoming Data packet in TCT. It is shown in Figure 6 that the $N_{1-L1}$ is unable to forward the Data packet towards the downstream node in the lower layer. However, the Data are received by the backup neighbors (e.g., $N_{5-L1}$, and $N_{3-L1}$). When the forwarder timer expires, the $N_{5-L1}$ forwards the Data towards the lower layer to avoid packet loss and retransmissions and to ensure minimal delivery delay. The upper layer node $N_{1-L2}$ purges the Data from TCT. This process continues until the Data successfully reach the consumer node.

#### 5.3.4. CIDF-WSN Cache Tables

To avoid retransmissions and minimize the packet losses in terms of both Interest and Data packets, CIDF-WSN proposes Interest Cache Table (ICT) and Temp Cache Table (TCT). The former avoids the interest packet failure and retransmissions when the nominated forwarder node fails to further relay the packet while the latter targets to minimize the Data packet failures in vulnerable network conditions. The detailed descriptions of both ICT and TCT are as follows.

1. **Interest Cache Table (ICT):** The Interest Cache Table (ICT) is maintained by the immediate neighbors of the designated forwarder node in the upper layer to avoid Interest Packet failure. The immediate neighbor(s) temporarily saves the incoming Interest packet in the ICT and forwards it if the designated node fails to further transmit the Interest Packet towards the potential data source.
2. **Temp Cache Table (TCT):** When the Interest packet arrives successfully at the destination of matching content, the provider node, after verifying the authenticity of Interest, sends back the requested content in the breadcrumb path. The breadcrumb path may crash and the requested content may be delayed or fail to reach the destined consumer. The delayed delivery of requested Data may highly degrade the network performance and it may cause havoc, as concerned departments may never take the action on time.
  To cope with the above-mentioned situation, CIDF-WSN develop a Temp Cache Table (TCT) which is enabled at the immediate neighbors of the designated forwarder node to ensure seamless communication and avoid packet losses in case the nominated node fails to perform the further Data packet transmissions. In this approach, when the node forwards the Data packet towards the designated node, the packet is also received by the immediate neighbors of the designated node due to shared medium. The immediate neighboring nodes keep these packets in their TCT, assign a forwarder timer to the packet, and wait for the transmission of the designated node. If the designated node fails to forward the packet, the neighbor node with the smallest forwarder timer value forwards the packet to streamline the communication process. In contrast, if the designated node successfully forwards the received Data packet, the upstream node(s) purges the TCT to allow future Data packet caching.
  It is worth mentioning that in both aforementioned Data forwarding modes, the TCT purge operation is always triggered when the downstream node forwards the data towards the consumer in the lower layer(s). The mechanism avoids TCT pollution and enables the node to allocate its TCT for future Data packets.

## 6. Performance Evaluation

In this section, we evaluate the trade-offs of CIDF-WSN via software-based simulations and compare the performance with the most recent state-of-the-art scheme developed for NDN-based WSNs—geographic interest forwarding (GIF) [27]. We also compare the performance of CIDF-WSN with Vanilla NDN [40] without performing any modification in the NDN codebase for WSNs. The detailed simulation environment and simulation results are depicted as follows.

### 6.1. Simulation Environment

In the simulations, CIDF-WSN consider several heterogeneous wireless sensor nodes deployed in the monitoring area of 100 m × 100 m with a total number of 100 nodes which are organized into five layers with an equal number of nodes. These nodes are static, and each node is equipped with a GPS module to acquire its physical location. The transmission range of nodes is restricted to one hop (i.e., towards the upstream node in the upper-layer or downstream node in the lower layer). The simulations are conducted in ndnSim [17] (an NS-3 extension developed for simulating NDN-enabled networks) on a desktop computer with a Core i5 CPU (2.90 GHZ), 8 GB of RAM, and a Linux Ubuntu operating system (i.e., 20.04).

The complete simulation parameters are summarized in Table 2.

### 6.2. Performance Evaluation Metrics

We considered the following evaluation metrics in our simulation study.

1. **Interest satisfaction rate (ISR):** The Interest satisfaction rate is a measure of the total number of Data packets $D_{pkt}$ successfully received in relation to the total number of Interest packets $I_{pkt}$ generated in the network. The high ISR corresponds to the high QoS of the network and vice versa. The mathematical formulation for ISR is presented as follows.
  $$\mathrm{Interest}\;\mathrm{satisfaction}\;\mathrm{rate}=\frac{\sum_{i=1}^{N}D_{pkt}^{i}}{\sum_{i=1}^{n}I_{pkt}^{i}}$$
2. **Interest satisfaction delay:** Interest satisfaction delay is defined as the time (*t*) taken by the Interest (I) to reach the provider (P), i.e., $t_{I,p}$, the Interest processing at the provider node $t_{I,p}^{P\prime }$, and the time taken by the Data packet to reach again the consumer node C, i.e., $t_{D,c}$.
  $$\mathrm{Interest}\;\mathrm{satisfaction}\;\mathrm{delay}=t_{I,p}+t_{I,p}^{P\prime }+t_{D,c}$$
3. **Total number of packets processed:** It corresponds to the total number of Interests and Data packets processed in the network.
4. **Total energy consumption:** The total energy consumption is defined as the amount of energy consumed by all the nodes (N) in Interest and Data packet forwarding.
  $$\mathrm{Energy}\;\mathrm{Consumption}=\sum_{i=1}^{N}e_{i}^{I_{pkts}}+e_{i}^{D_{pkts}}$$
5. **Communication overhead:** The communication load refers to the overall bandwidth consumption that may happen due to Interest or Data packet transmissions in the network.

### 6.3. Evaluation Results

The performance evaluation results are presented as follows.

#### 6.3.1. Interest Satisfaction Rate (ISR)

**Figure 7:**
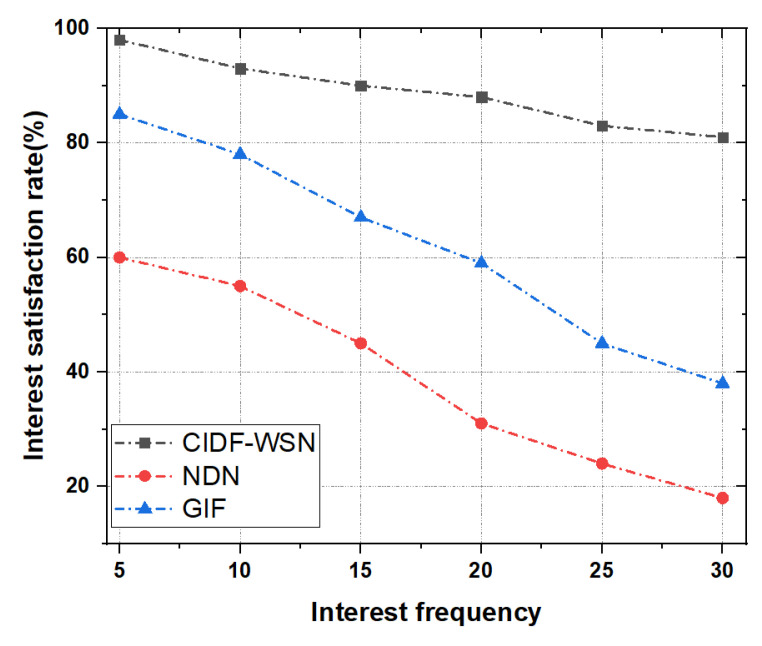
Interest satisfaction rate as a function of Interest.

To analyze the Interest satisfaction rate (ISR), CIDF-WSN vary the number of Interest packets between 5 to 30 Interest packets/sec. Figure 7 depicts the ISR as a function of Interest frequency in the network. It is evident from the results that the CIDF-WSN has a better ISR compared with both benchmark schemes. The graph shows that the CIDF-WSN achieves around 80% satisfaction rate in extreme conditions (e.g., 30 Interests/second) compared to the GIF; however, the GIF satisfied below 50% of generated Interests at the same rate. The rationale is that in the CIDF-WSN when the designated forwarder fails to make further transmissions—Interest or Data packet—the neighbors of the designated forwarder pointing towards the consumer (by consulting their ICT and TCT) collaborate and resume the transmissions to avoid communication failure. Moreover, the updated NFIB-based next forwarder selection and TCT-assisted Data packet forwarding play a key role in minimizin the Interest and Data packet losses and retransmissions. This significantly contributes to enhancing ISR.

In contrast, the GIF may involve several retransmissions in vulnerable conditions when the designated node fails to transmit the Interest packet. In addition, if the potential node fails to find the next-hop forwarder, it reinitiates the neighbor table development to assign the potential forwarder to the received Interest for further transmissions. All these factors induce congestion and collisions and increase the overall traffic in the network. In comparison with the vanilla NDN, the CIDF-WSN shows much better results. The minimized Interest and Data transmissions avoid the congestion in the network and increase the performance in terms of ISR. Whereas, in NDN the blind Interest and Data packets transmissions turn into the broadcast storm and collisions which, as a result, lower the ISR.

#### 6.3.2. Interest Satisfaction Delay

**Figure 8:**
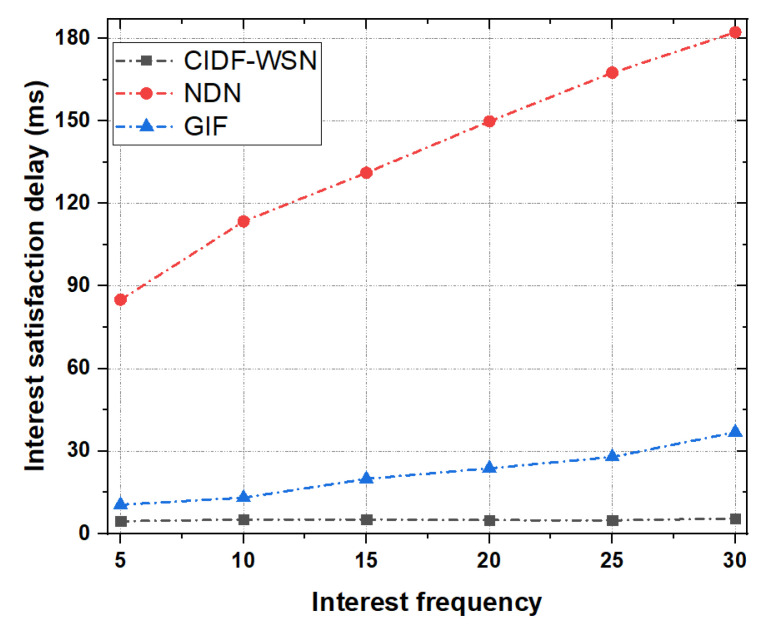
Interest satisfaction delay as a function of Interest.

Figure 8 shows the Interest satisfaction delay as a function of the number of Interest packets. To study the Interest satisfaction delay, we vary the Interest packet frequency between 5 and 30 packets/sec. The simulation results reveal that both NDN and GIF underperform compared with the CIDF-WSN in terms of Interest satisfaction delay(s). It is clear from the results depicted in Figure 8 that the CIDF-WSN achieves above 70% lower Interest satisfaction delay compared with the GIF while the amount of delay is several times lower in the case of NDN. The rationale is that the NFIB cost maintenance at every successful transmission of the upper-layer node also enables the lower-layer node to select the optimal node before Interest transmission to avoid the interest losses and retransmissions by the lower-layer node. Moreover, the proposed Interest and Data forwarding mechanism avoids the congestion and collisions in the network, which may hinder seamless communication. All these factors significantly contribute to reducing the Interest satisfaction delays. On the contrary, in both GIF and NDN, the lack of efficient Interest and Data forwarding mechanisms generates the extra network overhead in terms of packet retransmissions, induces congestion, and increases the overall Interest satisfaction delays.

#### 6.3.3. Total Number of Packets Processed

**Figure 9:**
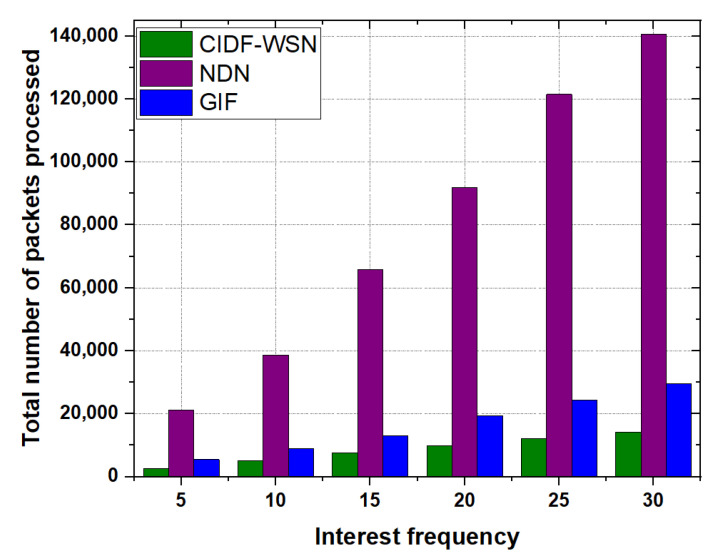
Total Packets processed as a function of Interest frequency.

The total number of packets processed as a function of the number of Interest packets is shown in Figure 9. The results show that the CIDF-WSN processed a far smaller number of packets—both Interest and Data packets—compared with GIF and NDN. It is taken into consideration that the proposed NFIB’s maintained cost and the robust Interest processing mechanism have a significant contribution in ensuring the less number of retransmissions. The former procedure helps in the selection of the optimal next-hop forwarder, whereas the latter enables the neighbor nodes to continue the communication process when the nominated forwarder fails to perform further transmission. This process assures the seamless end-to-end Interest and Data delivery process with the minimum number of transmissions, thus significantly minimizing the packets to be processed by each node in the network. In contrast, in the GIF, when the relay node fails to further transmit the packet, the forwarder restarts the whole process without considering the potential neighboring nodes to resume the communication process. Moreover, if there is no information of sender in the forwarder’s neighbor table, the forwarder re-initiates the neighbor table maintenance procedure. With the lack of immediate neighbor collaboration in packets transmissions and frequent neighbor table maintenance procedure, redundant packet transmissions increase the number of packets required to be processed, which, as a result, also affects the Interest satisfaction delays.

On the other hand, in NDN, each node rebroadcasts the received packet which significantly increases the overall transmissions in the network which in turn results in the wastage of network resources, congestion, and eventually increases the number of processed packets in the network.

#### 6.3.4. Total Energy Consumption

**Figure 10:**
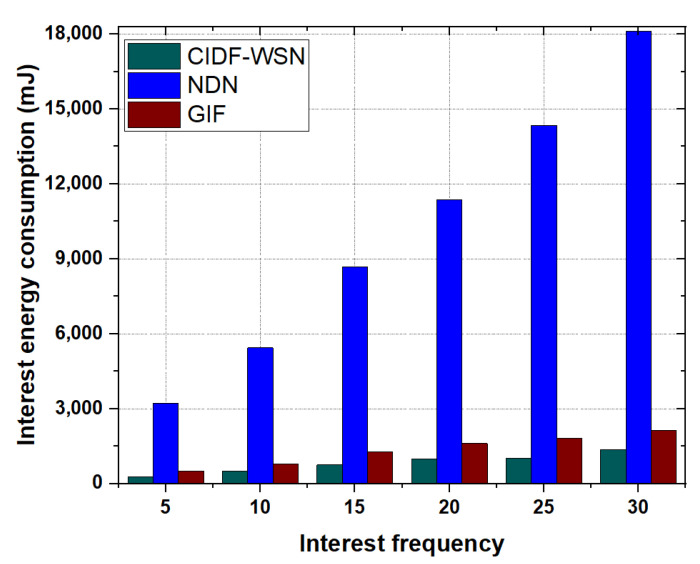
Interest energy consumption as a function of Interest.

**Figure 11:**
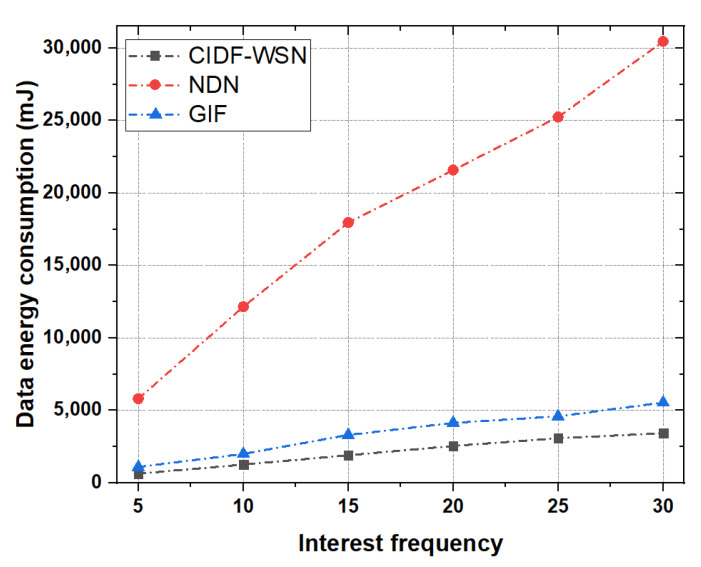
Data energy consumption as a function of Interest.

Wireless transmissions in terms of Interest and Data packet disseminations account for the majority of a node’s energy consumption. Figure 10 and Figure 11 show the comparative analysis of total energy consumed in Interest and Data packet transmissions in GIF, NDN, and CIDF-WSN.

1. **Interest Energy consumption:** To study the impact of Interest energy consumption, CIDF-WSN vary the Interest packet frequency between 5 and 30 packets/sec and compute the total Interest energy consumption of all schemes, as shown in Figure 10.
  It is evident from the results that the overall energy consumption in all the schemes increases with an increase in the Interest packet frequency. However, the CIDF-WSN lowers more than 50% of energy consumption compared with GIF, while reducing the energy consumption several times in comparison with the vanilla NDN. This is because that the CIDF-WSN avoids the blind Interest broadcasts by utilizing the ICT of the neighboring node(s) of the nominated relay to forward the Interest packet in vulnerable conditions. If the designated node fails to reply to the Interest packet, the immediate neighbors of the designated node forward the Interest packet by consulting their ICT to avoid the blind broadcast of the Interest packet. These procedures highly reduce the retransmissions and conserve the transmission energy of the node.
  In GIF, if the forwarder fails due to a certain reason, the content consumer restarts the Interest transmissions upon the Interest timeout. In addition, If a node receives an Interest packet and there is no information on sender in its neighbor table, the node re-initiates the neighbor table development procedure which involves multiple Interest packets transmissions which enhances the overall energy consumption of underlying nodes. Moreover, there is no procedure provided by the GIF which may allow the immediate neighbors of the selected relay to continue the Interest forwarding process in traffic-intensive conditions. All these factors result in increasing the total Interest energy consumption of the network. Analyzing the Interest energy consumption of vanilla NDN (shown in Figure 10) compared with the CIDF-WSN, the energy consumption of vanilla NDN increases with the increase in Interest frequency. In vanilla NDN, the recipient node(s) blindly forward the Interest packets if they do not have the requested content in their CS. This induces the Interest broadcast storm due to several Interest transmissions and increases the overall Interest energy consumption of the network.
2. **Data Energy consumption:** Data packets have a major impact on total energy consumption due to their large size. Figure 11 shows that in all the schemes, the energy consumed by the Data packets is greater than that of the Interest packets. The rationale is that the size of the Data packet is almost double that of the Interest packet. In comparison with vanilla NDN and GIF, the CIDF-WSN produced better performance results in terms of total Data packets energy consumption. The reason is that the robust Data forwarding mechanism allows the immediate neighbors of potential Forwarder nodes in the lower layer directing towards the consumer to temporarily utilize their TCT and continue the packet forwarding process in case Data packet transmission stops due to designated node failure.
  The collaboration of immediate neighbors in the Data packet forwarding process reduces the overall Data packet retransmissions and conserves the energy of the nodes. In contrast, the GIF starts over the entire communication process when the packet has failed to receive at the consumer end. Another important reason is that the lack of involvement of immediate neighboring nodes of the potential forwarder to carry the Data packet forwarding in case of designated node failure leads towards frequent packet retransmissions and consumes a substantial amount of energy. On the other hand, similar to the GIF, the vanilla NDN rebroadcasts the incoming Data packets to the downstream neighboring nodes. The frequent Data packet transmissions increase the overall energy consumption of the network which can be visualized in Figure 11.

#### 6.3.5. Communication Overhead

**Figure 12:**
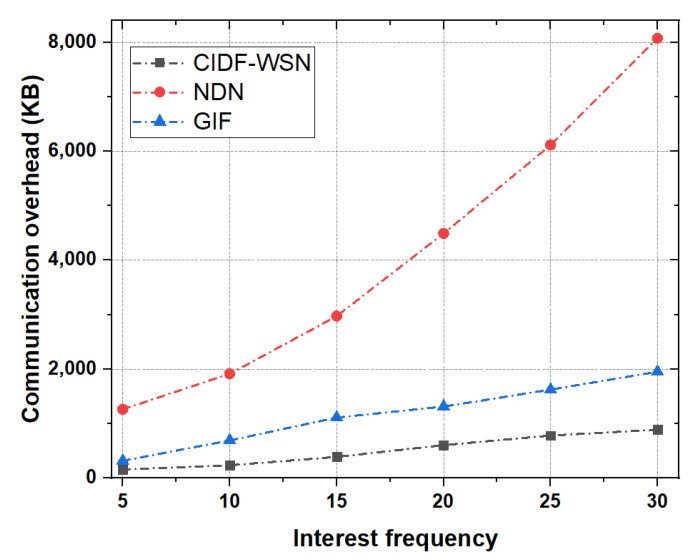
Communication overhead as a function of Interest.

Figure 12 shows the comparison results of communication overhead as a function of the number of Interest packets in the network. For the network overhead analysis, we vary the Interest generation frequency between 5 Interests/secs to 30 Interests/sec. It is evident from the results that the CIDF-WSN has a lower communication overhead compared to the NDN and GIF. It can be observed from the results that the network overhead in the CIDF-WSN is around 1000 KB against 30 Interests/sec while being extremely low in low-interest packet frequency (e.g., approximately 100 KB against 5 packets/secs). The main reason behind the reduced overhead is the proposed Interest and Data transmissions mechanism accompanied by the corresponding Cache table, which significantly reduces the number of Interest and Data packet retransmissions. As already expressed in the previous results, the ICT and TCT tables are utilized by the immediate neighbors during the vulnerable network conditions when the designated nodes fails to perform further transmissions. The proposed procedure avoids the packet retransmissions by the downstream forwarder and significantly reduces the communication overhead. However, in both GIF and NDN, the communication overhead highly increases with the increase in interest frequency. GIF generates more than the double overhead packets while in the NDN this figure is significantly greater compared with both GIF and CIDF-WSN. In NDN, each node rebroadcasts the packet in case of a cache miss, while in GIF the number of retransmissions increases when a node fails to retransmit the packet further due to its resource constraints and vulnerable channel conditions. All these factors significantly contribute to increasing the overall communication overhead and affect the entire performance of the network.

## 7. Conclusions and Future Work

In this paper, we propose a new next forwarder selection and an efficient Interest and Data transfer mechanism for single radio WSNs. CIDF-WSN develop a NFIB table for optimal next-hop selection. In addition, to reduce the interest packets loss and Interest retransmissions, an efficient Interest packet transmission mechanism based on ICT is proposed. Furthermore, to avoid Data packet losses, we devise a robust Data recovery mechanism accompanied by the TCT. The experimental results confirm that the CIDF-WSN magnifies the ISR, and minimizes the Interest satisfaction delay, the communication overhead, and total energy consumption of the network.

In future work, CIDF-WSN aims to include the mobility aspect and analyze the performance in the mobile environment by simulating various large and complex scenarios. Moreover, we will adopt AI schemes for optimal cache decisions regarding ICT and TCT mechanisms.

## Figures and Tables

**Table 1:** Summary of Related Work.

Ref	Title	Packet Recovery	Downstream Processing	NFIB Update	Layer Arch
[21]	CCN-WSN	×	×	×	×
[22]	DMIF	✓	×	✓	×
[23]	BLOOGO	×	×	✓	×
[24]	CCN Caching	×	×	×	×
[25]	Muti source DataRetrieval	✓	×	×	×
[26]	NETWRAP	×	×	×	×
[27]	GIF	✓	×	✓	×
Proposed	CIDF-WSN	✓	✓	✓	✓

**Table 2:** Simulation Parameters.

Parameter	Value
Simulator	NS-3 (ndnSIM 2.5)
Area	100 × 100
Total number of sensor nodes	100
Node distribution	layered distribution
Communication stack	NDN
Wireless interface	IEEE 802.15.4
Propagation delay model	Constant Speed Propagation Delay Model
Energy consumption	0.5 μJ/bit
PIT Timer	4 sec
Interest Packet size	48 bytes
Data Packet size	96 bytes
Simulation time	1800 sec
